# Pollution, Exposure and Risk of Biogenic Amines in Canned Sea Fish: Classification of Analytical Methods Based on Carbon Spheres QuEChERS Extraction Combined with HPLC

**DOI:** 10.3390/molecules27196243

**Published:** 2022-09-22

**Authors:** Xinying Guo, Zhiying Dai, Weibing Zhang

**Affiliations:** 1 Nantong Center for Disease Control and Prevention, Nantong 226001, China; 2 Nantong Key Laboratory of Food Hygiene, Nantong Food Safety Testing Center, Nantong 226001, China; 3 Nantong Teaching and Research Practice Base of Public Health and Preventive Medicine of School of Public Health, Lanzhou University, Nantong 226001, China

**Keywords:** fish products, biogenic amines, QuEChERS, food safety index, pollution characteristics, exposure

## Abstract

This study investigated the pollution characteristics, exposure levels and health risk assessments of seven kinds of biogenic amines (BAs) in eight varieties of canned sea fish products (*n* = 131) on the Chinese market. Carbon spheres QuEChERS mixed dispersion solid phase extraction combined with HPLC was used for the classification and analysis of batch samples. The average recovery of single BAs obtained by this method is 92.3~97.7%, and the relative standard deviation is 1.9~4.8%. Different varieties of samples have different degrees of pollution, the mass concentration of single BAs range 0.45~27.74 mg/kg, and the total concentration of ΣBAs range 18.77~368.50 mg/kg, of which the concentration of Σ4BAs range 11.53~368.50 mg/kg. The composition of four BAs is mainly putrescine, cadaverine, histamine and tyramine, which always play an important role in the exposure level and risk assessment of samples. The exposure level of BAs in the human body ranges 67.03~209.52 μg∙kg^−1^∙d^−1^. The health risk assessment shows that the gender trend of exposure risk level of BAs is male > female (young age), female > male (middle and old age), the age trend is young age > old age > middle age, and the regional trend is city > countryside. The food safety index of BAs in samples is 0.0062~0.0195, which is far less than 1, so the risk is within the controllable range.

## 1. Introduction

Biogenic amines (BAs) is a general term for a class of nitrogen-containing aliphatic, aromatic or heterocyclic organic compounds generated by the decarboxylation of amino acids or the transamination of aldehydes and ketones by enzymes in microorganisms. Biogenic amines have low molecular weight and polar or semi polar characteristics. As hormones or neurotransmitters, they are an important biological function, participate in local immune response, inflammatory response, and regulate intestinal physiological movement. Moderate intake of BAs can regulate the physiological activities of healthy people [1,2]. However, if the accumulation of BAs in the human body reaches a high level, or if there is excessive intake, it will cause irreversible harm to human health [3,4]. If the limit is higher, it will damage the human nervous and cardiovascular systems and other organs, and will also lead to headaches, nausea, respiratory distress, palpitations, vomiting, diarrhea and many other adverse, even life-threatening, symptoms. As a kind of low molecular weight compound with biological activity and containing amino groups, BAs have widely existed in various animal and plant tissues and their food products, especially in protein rich aquatic products, such as marine fish and canned products. Studies have reported that high histamine fish, such as tuna, mackerel, saury and other fish and their products, are one of the foods with the highest content of biogenic amines [5,6]. The fermented meat will produce a large number of BAs in the production and circulation process. The nitrite added in the production process of salami reactions will react with BAs in the stomach acid environment to produce a kind of carcinogenic and carcinogenic substance nitrosamine, thus increasing carcinogenic toxicity and disease burden. At present, some countries have tried to provide the limit standards of BAs according to the characteristics of different food. The United States stipulates that the histamine content in aquatic products and their products shall not exceed 50 mg/kg; the European Union stipulates that the content of histamine in food shall not exceed 100 mg/kg, and the content of tyramine shall not exceed 100~800 mg/kg; China stipulates that the histamine content in mackerel shall not exceed 1000 mg/kg, and that in other marine fish shall not exceed 300 mg/kg. Nowadays, canned sea fish has become a fast-selling food and is resident on the table of consumers because it is delicious, instant and portable. However, due to the limited production process and storage technology, the internal reaction will produce BAs. These will have a huge impact on quality and safety without appropriate controls. Therefore, content detection of histamine, tyramine and other BAs in canned sea fish has become an important indicator of food quality and safety [7,8].

At present, in the relevant studies on BAs in canned sea fish at home and abroad, high performance liquid chromatography (HPLC) and liquid chromatography mass spectrometry (LC-MS) has become the most frequently used method for the determination of biogenic amines. LC-MS [9,10] pretreatment is relatively simple, highly sensitive, and does not require derivatization. However, the instrument itself is expensive, so it is difficult to promote at the grass-roots level. In contrast, HPLC has shown its advantages. It can meet the demands of most samples in terms of test accuracy and detection limits and is easier to promote at the grass-roots level [11,12]. In terms of detector selection, the UV detector only needs the target compound to have the appropriate chromogenic group and determine the appropriate UV spectral wavelength for analysis, while mass spectrometry must provide mass to charge ratio information, and multi-stage mass spectrometry also needs to provide mass to charge ratio information of compound fragments. Although the sensitivity of mass spectrometry is higher than that of UV, mass spectrometry generally requires that compounds can be ionized, and mass spectrometry detection also requires more stringent requirements for the front-end liquid phase mobile phase and chromatographic column. The acquisition cost and maintenance cost are higher than those of UV detectors, which is not conducive to the promotion of grass-roots units. Therefore, in the detection of biogenic amines, it is more appropriate to use ultraviolet detector, which is also more favored by researchers. In terms of pretreatment methods, the main ones are solid-phase extraction [13] and pre-column derivatization [14,15,16], but the traditional solvent extraction method requires not only a large number of organic solvents, but also repeated extraction, degreasing, purification and concentration steps, which are cumbersome, time-consuming and laborious, and extremely environmentally friendly. Pre-column derivatization requires a series of processes, such as extraction, purification, degreasing and impurity removal in combination with samples, and the derivatization steps are meticulous and complex. The selection of derivatization agent, derivatization dose, derivatization temperature, derivatization time and other factors have a great impact on the derivatization effect [17,18]. Dansyl chloride [19] and the o-phthalaldehyde [20] pre-column derivatization method are most commonly used in food pretreatment. However, the substrate of fish products is complex, and the efficiency of the reagent derivatization alone cannot be guaranteed. In addition, the BAs in the sample are easy to decompose and the content is minimal, so the final detected BAs content may be different from the actual content. Therefore, it is necessary to develop sample pretreatment methods that are simple and easy to operate, reliable, environmentally friendly, and have high derivation efficiency [21,22]. In the risk assessment, there are some reports in recent years, such as Magro et al. [23], on the histamine pollution and exposure assessment report of fish products in southern Italy. Afé et al. [24] analyzed the BAs content of smoked fish in Benin, Africa, and put forward food safety suggestions to consumers exposed to elevated concentrations. Koral et al. [25] investigated the BAs content and some food safety parameters of salted fish products in Turkey and European countries, which pointed out the comprehensive factors causing health risks. However, there are few research reports on the human exposure level and risk assessment of various BAs in different marine fish and their products sold on the Chinese market, and there is no comprehensive quantitative exposure level and food safety risk assessment of people in different regions, different sexes and different age stages. Therefore, in order to ensure the safety of aquatic products and effectively control BAs, researchers need to conduct more in-depth research on the pollution level of BAs and human health. On the one hand, researchers need to find more simple, sensitive and cheap detection methods and improve the online rapid quantitative detection technology for BAs. On the other hand, it is urgent to comprehensively evaluate the intake risk, population health and control management of BAs, so as to guarantee the quality and safety of aquatic products, such as marine fish products and consumer food safety [26].

Aiming at the problems in the existing technology, such as the complex substrate of canned sea fish, the low efficiency of direct reagent derivatization, and the decomposition of some BAs caused by the long pretreatment time of samples, this study provides a new dispersed solid-phase extraction adsorbent carbon spheres for the determination of BAs in canned sea fish. Carbon spheres are mainly aimed at the rapid pretreatment of samples with complex substrates, with high derivatization efficiency and good selectivity. It can accurately and efficiently realize the simultaneous quantitative determination of various BAs in different canned sea fish samples. At the same time, batch data were statistically analyzed to examine the human exposure level and food safety risk assessment of canned sea fish. According to the dietary consumption data in the monitoring report on the nutrition and health status of Chinese residents from 2010 to 2013, the population with differences in different regions, sexes and age stages was quantified, and the dietary exposure distribution characteristics and health risks of canned sea fish products were comprehensively evaluated. This research results can provide a theoretical reference for exploring and optimizing the formulation of a more reasonable standard for the content of BAs in aquatic products, and the government supervision agencies to formulate scientific and effective supervision.

## 2. Results and Discussion

### 2.1. Morphological Characterization of Carbon Spheres

**Figure 1:**
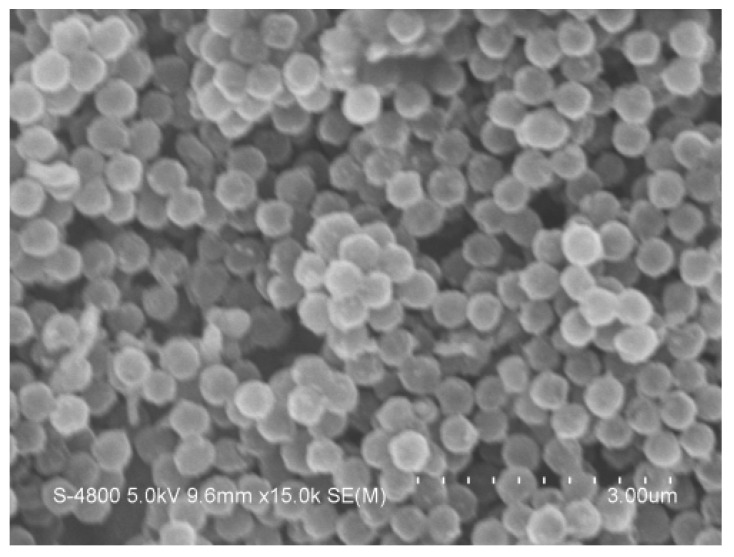
SEM images of carbon spheres.

Analysis of phenolic resin based carbon spheres (PFC/CS) was carried out on scanning electron microscopy (SEM). As illustrated in Figure 1, PFC/CS was nanoscale in size, the diameter of carbon sphere was about 320 nm, with a spherical structure with good dispersion, which could provide large specific surface areas as interaction sites.

### 2.2. Optimization of Sample Preparation

#### 2.2.1. Sample Extraction

**Figure 2:**
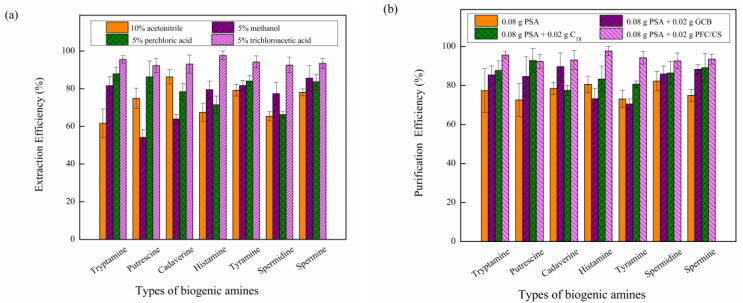
(**a**) Extraction effect of four organic solvents, 10% acetonitrile, 5% methanol, 5% perchloric acid and 5% trichloroacetic acid. (**b**) Purification effect of four adsorbents, 0.08 g PSA, 0.08 g PSA + 0.02 g GCB, 0.08 g PSA+ 0.02 g C_18_, and 0.08 g PSA+ 0.02 g PFC/CS.

In order to ensure the effectiveness of sample extraction and the quality control of the test process, four different organic solvents (a) 10% acetonitrile, (b) 5% methanol, (c) 5% perchloric acid, and (d) 5% trichloroacetic acid were used to extract homogeneous samples, and the extraction effect of organic solvents was investigated. As shown in Figure 2a, less impurities were extracted from aqueous acetonitrile, but the recovery rate of tryptamine was low. After methanol was mixed with a certain proportion of water, the impurities extracted were higher, and the recovery rate of putrescine and cadaver was lower, which affected the accuracy of quantitative analysis. A total of 5% perchloric acid can maintain the weak acidic extraction environment, but the recovery rate of histamine and spermidine extracted is low. Moreover, 5% trichloroacetic acid shows higher extraction efficiency than 5% perchloric acid, which may be because trichloroacetic acid, as an organic acid, is more conducive to the extraction of biogenic amines than strong acidic inorganic acid at weak acidic pH. This conclusion is consistent with our previous research results [27].

#### 2.2.2. Sample Cleanup

Commonly used adsorbents include propyl ethylenediamine (PSA), octadecylsilane (C_18_) and graphitized carbon black (GCB). PSA adsorbent can remove many polar interference components, such as fatty acids, lipophilic pigments and sugars, but the effect of removing sterols is general. GCB adsorbent has a good effect on removing cholesterol, sterols and pigments, but the dosage is exquisite when adsorbing aromatic amines. Excessive amount will lead to a sharp drop in the recovery rate. C_18_ can absorb impurities, such as lipids, pigments and aroma components, which are often mixed with PSA. In this study, the modified QuEChERS method was used to remove water with anhydrous sodium sulfate, and (a) 0.08 g PSA, (b) 0.08 g PSA + 0.02 g GCB, (c) 0.08 g PSA + 0.02 g C_18_, and (d) 0.08 g PSA + 0.02 g PFC/CS mixed adsorbents were used to investigate their purification effects, shown in Figure 2b. The mixed packing of PSA and PFC/CS can significantly remove the interfering impurities in the extraction solution, with the highest recovery rate, the best purification efficiency and the best purification effect.

### 2.3. Method Validation

#### 2.3.1. Linearity and Sensitivity

**Figure 3:**
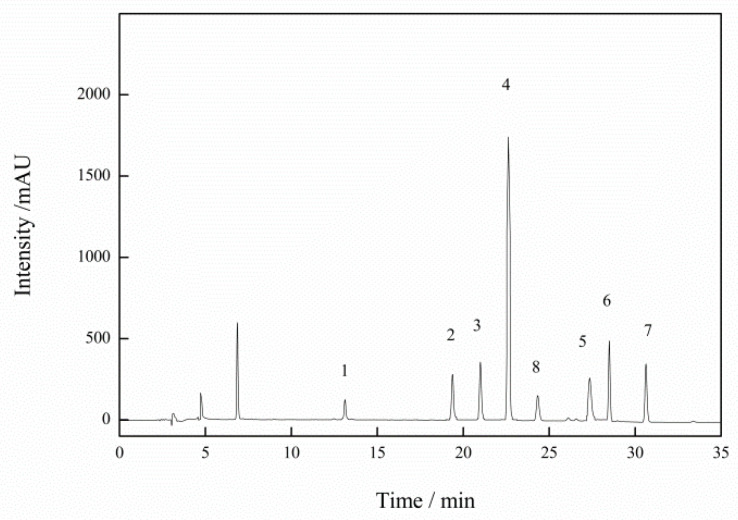
Chromatogram obtained by HPLC at 254 nm of biogenic amine standard solutions at 50 µg/mL and the internal standard (IS) after derivatization. The components are: 1. tryptamine; 2. putrescine; 3. cadaverine; 4. histamine; 5. tyramine; 6. spermidine; 7. spermine; 8. 1,7-diaminohepane (IS).

Figure 3 shows the chromatogram of analytical standards of seven kinds of BA standard solutions. The limit of detection (LOD) and the limit of quantification (LOQ) were defined as signal-to-noise ratios of 1/3 and 1/10 (*n* = 6). The results showed that the detection limit (LOD) of the seven BAs ranged 7.2~10.8 mg/kg, the limit of quantitation (LOQ) ranged 24~36 mg/kg, and the correlation coefficient (R^2^) was 0.9996~1. Compared with China’s current national food safety standard (Determination of biogenic amines in food GB 5009.208-2016), the detection limit is lower than the standard value (50 mg/kg), and the method performance is better than the current national standard, with wide linear range and high linear coefficient.

#### 2.3.2. Precision and Recovery

The precision of the method was calculated by measuring the spiked BA samples with low, medium and high concentrations. The recovery rate is calculated by calculating the ratio of the actual mass concentration to the spiked concentration according to the standard curve equations. Adding the BAs mixed standard solution to representative samples, the average recovery of low, medium and high concentrations is 92.3~97.7%, and the relative standard deviation RSD (%) is 1.9~4.8%. See Table 1 for specific results. The method has high recovery and good precision and can be used for the determination of biogenic amines in canned fish products.

### 2.4. Health Risk Assessment Model

#### 2.4.1. Assessment of Dietary Exposure

In order to reflect the principle of protecting the majority of the population, this study used the point assessment model to analyze the dietary exposure level of canned sea fish in the human body and took exposure screening as the first step of the exposure assessment. It is intended to assume that hazard intake is equal to a fixed value of food consumption (e.g., average or higher consumption data) multiplied by the residual content or concentration of the hazard (the average residual level or upper limit of the legally permitted value). Formula (1) was used to calculate dietary exposure levels:

(1) $$y=\sum_{n}^{M}\frac{x_{n,97.5}\;\cdot \;C_{n,max}\;\cdot \;DI\;\cdot f\;\;}{W\;}\;$$

where *x_n_*_,97.5_ representing the consumption distribution range of the nth food is 97.5%, *C_n_*_,_*_max_* represents the maximum residue of biogenic amines in the *n*th food (mg/kg), *M* represents the type of food consumed by people, *DI* represents the daily average intake of people (g/d), *f* represents the conversion coefficient (×10^−3^), *W* represents the average weight of the population (kg), and *y* represents the exposure of BAs in the population (μg∙kg^−1^∙d ^−1^).

#### 2.4.2. Food Safety Index Evaluation

In order to improve the reliability, simplicity and identifiability of food safety, the deterministic hazards of substances in food are taken as the evaluation object, the food safety limit standard is taken as the fundamental basis, and the risk assessment and early warning model “food safety index (IFS*c*, also known as the food safety early warning index)” is taken as a weightless food safety risk evaluation index. Formulas (2)~(4) are used to calculate the food safety index:

IFS*c* = XS*c*/(*W*·SI*c*) (2)

XS*c* = λ·EDI*c*·*f* (3)

EDI*c* = ∑R*i*·DI*i* (4)

where λ is the digestion and absorption rate of pollutant *C* in food *i* in the human body; XS*c* is the actual digestion and absorption of pollutant *C* in food *i* in the human body (mg/d); *f* is the conversion factor (×10^−5^). EDI*c* is the estimated value of the actual daily intake of chemical component *C*, R*i* is the residue of pollutant *C* in food *i* (mg/kg); DI*i* is the estimated daily intake of food *i* (g/d), and SI*c* is the maximum tolerance of the human body to BAs (mg/(kg·bw)).

### 2.5. Samples Pollution Levels

**Figure 4:**
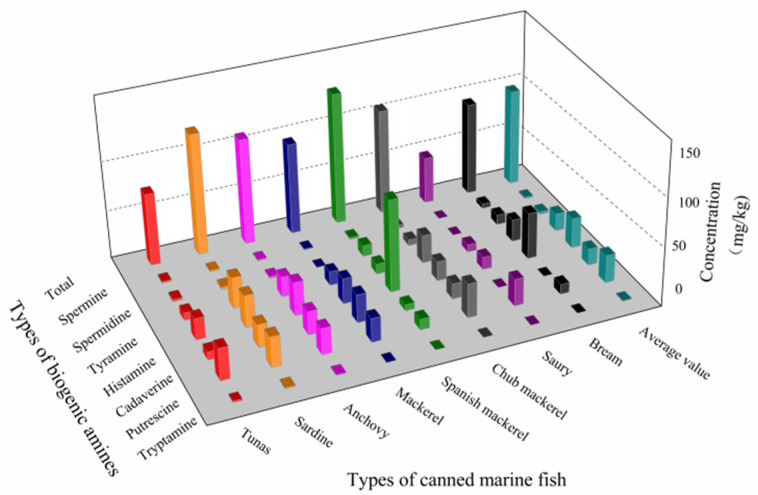
Mass concentration of 7 biogenic amines in 8 kinds of canned sea fish.

**Figure 5:**
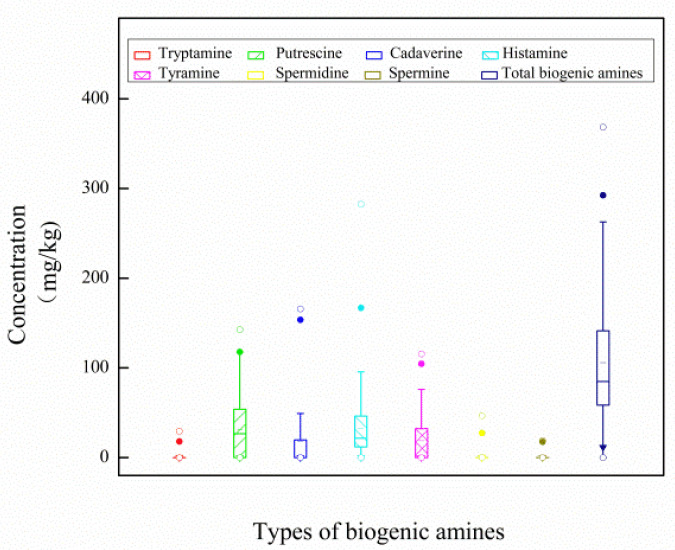
Box diagram of concentration distribution of 7 biogenic amines.

**Figure 6:**
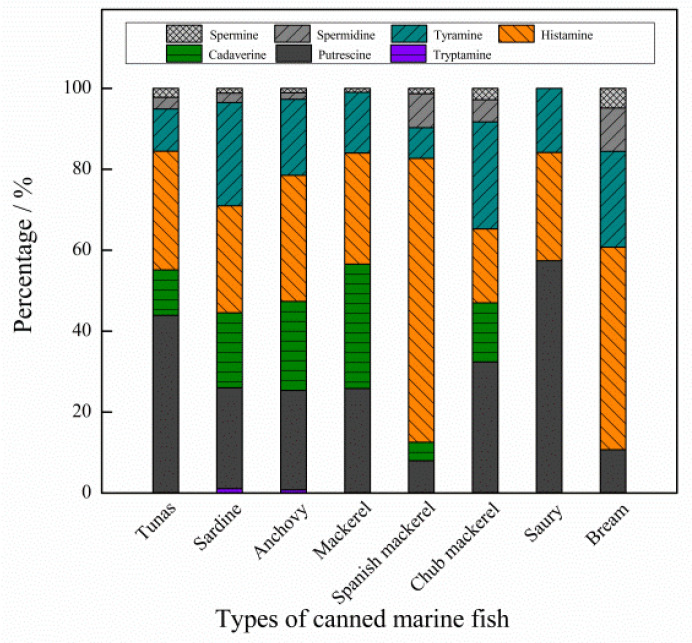
Percentage of 7 kinds of biogenic amines.

The mass concentration distribution of seven BAs in eight kinds of canned sea fish is shown in Figure 4. Among all samples, the histamine content of Spanish mackerel is the highest, with an average of 85.44 mg/kg and a concentration range of 29.40~282.50 mg/kg. The second is bream, with the average value of the sample being 42.67 mg/kg, and the concentration range being 12.00~82.36 mg/kg. A box diagram of the mass concentration of seven kinds of BAs is shown in Figure 5, and the order of BAs content in eight kinds of canned sea fish samples is histamine (His, 27.74 mg/kg) > putrescine (Put, 25.81 mg/kg) > tyramine (Tyr, 16.25 mg/kg) > cadaverine (Cad, 15.14 mg/kg) > spermidine (Spd, 2.44 mg/kg) > spermine (Spm, 1.34 mg/kg) > tryptamine (Try, 0.45 mg/kg). The percentage of seven BAs shows that Σ BAs4 (Put, Cad, His and Tyr) accounts for more than 84.46% of the total BAs, as shown in Figure 6, and the average percentage of all BAs is His (34.95%) > Put (28.48%) > Tyr (17.92%) > Cad (12.70%) > Spd (3.93%) > Spm (1.78%) > Try (0.23%).

The data in Table 2 show that the histamine content of some samples exceeds the histamine content in aquatic products specified in the United States (50 mg/kg), which is ranked as Spanish mackerel (50.00%) > bream (33.33%) > anchovy (29.17%) > mackerel (21.43%) > chub mackerel (20.00%) > sardine (18.18%) > tunas (9.52%) according to the excess rate, but it did not exceed the Chinese national standard (400 mg/kg) for high histamine fish, nor did it exceed the histamine content (100 mg/kg) specified by the European Union. The tyramine content of 6.06% sardine samples exceeded the EU recommended value (100 mg/kg), but from the average value, it can be seen that the average value of single component biogenic amines in all samples exceeding the limit (except one Spanish mackerel sample, 52.64 mg/kg) did not exceed 50 mg/kg.

Table 3 lists the detection rates of eight kinds of canned sea fish. The detection ranges are tunas (ND~59.52%), sardine (6.06~72.73%), anchovy (4.17~87.50%), mackerel (ND~57.14%), Spanish mackerel (ND~100%), chub mackerel (ND~60.00%), saury (ND~25.00%), and bream (ND~100%). At the same time, the detection ranges of seven BAs were Try (ND~6.06%), Put (25.00~57.14%), Cad (ND~35.71%), His (25.00~100%), Tyr (14.29~66.67%), Spd (ND~33.33%), and Spm (ND~33.33%).

### 2.6. Samples Risk Assessments

#### 2.6.1. Dietary Exposure Assessment

**Figure 7:**
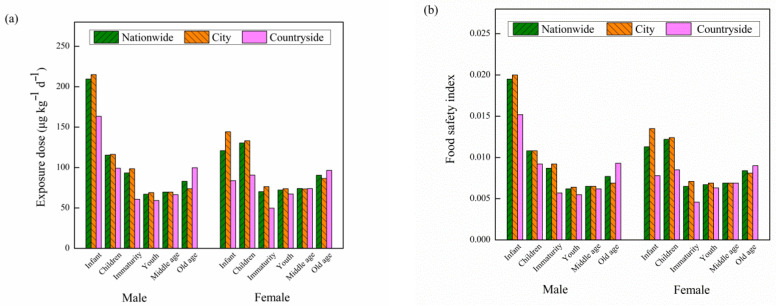
Dietary exposure (**a**) and food safety index (**b**) of total biogenic amines in different regions, sexes and age stages.

According to the survey results and the weight of urban and rural residents and the per capita dietary consumption of aquatic products in the monitoring report on the nutrition and health status of Chinese residents from 2010 to 2013, the exposure of different kinds of BAs in different regions, sexes and age stages can be obtained by substituting them into Formula 1, as shown in Table 4. It can be seen from different regions that the daily exposure of total biogenic amines per person nationwide ranges 67.03~209.52 μg·kg^−1^·d^−1^, of which the exposure of urban residents ranges 69.08~214.99 μg·kg^−1^·d^−1^, and the exposure range of rural residents is 49.81~163.42 μg·kg^−1^·d^−1^. At different age stages, the exposure ranges of young people (2~17 years old) in nationwide, city and countryside residents are 70.19~209.52 μg·kg^−1^·d^−1^, 76.44~214.99 μg·kg^−1^·d^−1^ and 49.81~163.42 μg·kg^−1^·d^−1^, respectively. The exposure ranges of middle-aged people (18~59 years old) in nationwide, city and countryside residents are 67.03~74.09 μg·kg^−1^·d^−1^, 69.08~73.86 μg·kg^−1^·d^−1^ and 59.33~73.98 μg·kg^−1^·d^−1^, respectively. The exposure ranges of elderly people (>60 years old) in nationwide, city and countryside residents are 83.04~90.45 μg·kg^−1^·d^−1^, 73.87~86.49 μg·kg^−1^·d^−1^ and 96.47~99.86 μg·kg^−1^·d^−1^, respectively. In terms of gender, male exposure ranges 59.33~214.99 μg·kg^−1^·d^−1^, and the female exposure range is μg·kg^−1^·d^−1^. Generally speaking, the characteristics of exposure level are city > countryside, young people > elderly people > middle-aged people, male > female, see Figure 7a.

#### 2.6.2. Food Safety Index Evaluation

The “food safety index (IFS*c*)” risk assessment and early warning model of different kinds of BAs in different regions, different sexes and different age groups is used as a weightless food safety risk assessment index, see Figure 7b. In different regions, the IFS*c* is 0.0062~0.0195 nationwide, 0.0064~0.0200 in cities and 0.0046~0.0152 in the countryside. At different age stages, the IFS*c* of young people (2~17 years old) in nationwide, city and countryside is 0.0065~0.0195, 0.0071~0.0200, 0.0046~0.0152, respectively, and the IFS*c* of middle-aged people (18~59 years old) is 0.0062~0.0069, 0.0064~0.0069, 0.0055~0.0069, respectively. The IFS*c* of >60 years old was 0.0077~0.0084, 0.0069~0.0081, 0.0090~0.0093, respectively. In terms of gender, the IFS*c* of male and female is 0.0055~0.0200 and 0.0046~0.0135, respectively. The results show that the biogenic amine content of some samples exceeds the domestic and foreign standards, but the food safety index is less than 1, so the risk is within the controllable range. At present, the international risk assessment of Bas in aquatic products mainly focuses on histamine, and other biogenic amines are rarely reported. A large number of studies have confirmed that biogenic amines, such as cadaverine, putrescine and tyramine, also exist in marine fish products. There is no unified standard for the safe limit of BAs at home and abroad, and only some regulations and suggestions are given for histamine and tyramine in some food. Overall, the characteristics of the IFS*c* are consistent with the exposure level. The IFS*c* is far less than 1, so the risk is within the controllable range.

## 3. Materials and Methods

### 3.1. Chemicals and Reagents

Histamine hydrochloride, tyramine hydrochloride, tryptamine hydrochloride, cadaverine hydrochloride, putrescine hydrochloride, spermidine hydrochloride and spermine hydrochloride standards (purity > 98.0%), internal standard 1,7-diaminoheptane and dansyl chloride derivative (purity > 99.0%) were purchased from Shanghai Anpel Reagent Co., Ltd. (Shanghai, China). Acetic acid, acetonitrile, and ammonium acetate were of GC grade and of highest purity (≥96.0%), and were purchased from Merck (Darmstadt, Germany). Hydrochloric acid, ammonia, sulfuric acid, nitric acid, acetone, ether, n-butanol, chloroform, phenol, resorcinol, sodium hydroxide and sodium bicarbonate were all purchased from Shanghai Sinopharm Chemical Reagent Co., Ltd., China. Ultrapure water was produced using the Milli-Q^TM^ advantage A10 purification water system (Madrid, Spain). Centrifuge (primo B, Thermo Fisher Scientific (China) Co., Ltd., Dreieich, Germany). Water bath pot (XMTD-8222, Shanghai Jinghong Experimental Equipment Co., Ltd., Shanghai, China). Ultrasonic cleaner (HN10-300D, Shanghai Hannuo Instrument Co., Ltd., Shanghai, China).

### 3.2. Sample

All canned sea fish (*n* = 131) were purchased from the market, the samples were divided into 8 categories: tunas (*n* = 42), sardine (*n* = 33), anchovy (*n* = 24), mackerel (*n* = 14), Spanish mackerel (*n* = 6), chub mackerel (*n* = 5), saury (*n* = 4), and bream (*n* = 3). A total of 500 g of the edible part from the canned sea fish was taken and stirred by a hand homogenizer, the sample was homogenized, placed into sterile plastic bags, and stored at −18 °C prior to further preparation and extraction.

### 3.3. Preparation of Standard Solutions

The quantification of BAs was performed by internal standardization, using 1,7-diaminoheptane at 200 mg/L in 0.1 mol/L hydrochloric acid solution as internal standard (IS). To prepare the calibration curve, 0.010 g of each biogenic amine standard was added into 0.1 mol/L hydrochloric acid solution to dissolve and fix the volume to 10 mL (1000 mg/L of single component biogenic amine). Then, 1.0 mL of each 1000 mg/L BAs was diluted to 100 mg/L in a hydrochloric acid solution (100 mg/L of mixed biogenic amine). Aliquots of 10 μL, 25 μL, 50 μL, 100 μL, 150 μL and 250 μL of the mixed biogenic amine standard solution were mixed with 250 μL of IS and diluted with 0.1 mol/L hydrochloric acid solution to a final volume of 1.0 mL, obtaining concentrations of 1.0 mg/L, 2.5 mg/L, 5.0 mg/L, 10.0 mg/L, 15.0 mg/L, 25.0 mg/L and 50.0 mg/L of biogenic amine.

### 3.4. Preparation of Phenolic Resin Based Carbon Spheres (PFC/CS)

The synthesis method is based on references [28] and [29]. A total of 0.1 mL of ammonia (25%) and 8.0 mL of absolute ethanol was dissolved in 20 mL of pure water, mixed at room temperature and stirred for 30 min. Next, 0.2 g phenol and 0.2 g resorcinol were dissolved and stirred at room temperature for 30 min, and 0.5 mL formaldehyde (37%) was added into a constant temperature water bath at 25 °C for 24 h. Next, the above solution was put into a hydrothermal reactor, kept at 150 °C for 3 h and then cooled. The reaction products were filtered and washed repeatedly with water and alcohol, centrifuged, and dried at 100 °C to obtain phenolic resin microspheres (PFC).

PFCs were heated at 150 °C, crosslinked and cured, placed in a high-temperature tubular furnace under the protection of nitrogen, and calcined at 800 °C for 3 h to obtain phenolic resin based carbon spheres (PFC/CS).

Next, 2.0 g PFC/CS powder was dissolved into 50 mL of sulfuric acid and 50 mL of nitric acid after ultrasonic oscillation and dispersion for 10 min, reflowed in a boiling water bath at 100 °C for 1 h, cooled to 50 °C and reflux was continued for 10 h, then cooled and the sediment was washed with water repeatedly to neutral, then ground into uniform fine powder after vacuum drying to obtain PFC/CS with oxidized surfaces. Scanning electron microscopy (SEM) was carried out on a Hitachi S-4800 scanning electron microscope (Tokyo, Japan) at an accelerating voltage of 5.0 kV and an FEI to ensure the successful synthesis of sample materials.

### 3.5. Sample Preparation and Pre-Column Derivatization

#### 3.5.1. Extraction

A total of 10 g of homogenized sample was put into a 100 mL conical flask with a stopper, and 250 μm of homogenized sample was added and fully mixed with the sample. Then, 40 mL of 5% trichloroacetic acid solution was added and extracted for 25 min in an ultrasonic bath. The extracted material was transferred to a 50 mL centrifuge tube and centrifugated for 20 min at 5000 rpm. The supernatant was transferred to another 50 mL centrifuge tube containing 0.5 g sodium chloride and 10 mL n-hexane. This tube was vortexed for 10 min, and the organic phase collected. If the emulsification occurred, centrifugal demulsification was carried out at 5000 rpm for 20 min.

#### 3.5.2. Purification

A total of 1.0 mL of organic phase was transferred and 0.02 g of PFC/CS and 0.08 g of n-Propyl ethylenediamine (PSA) solid adsorbent was added and mixed into a covered micro centrifuge tube, vortexed violently for 2 min, fully dispersed and purified, centrifuged at 5000 rpm for 15 min, and then the supernatant sucked to be derived.

#### 3.5.3. Derivatization

The derivatization procedure of samples was improved on the basis of reference [19]. First, 1.0 mL of the above supernatant was derived and 1.0 mL of saturated sodium bicarbonate solution, 20 μL 5.0 mol/L sodium hydroxide solution and 1.0 mL 10 mg/mL dansyl chloride derivatization reagent were added, mixed and placed in a constant temperature water bath at 60 °C for a derivatization of 45 min. Next, 100 μL ammonia water was added, followed by 0.5 g sodium chloride and 5 mL ether, fully vibrated for 10 min and dried with nitrogen in a water bath at 35 °C. Then, 1.0 mL of acetonitrile was dissolved and filtered with 0.22 μm organic membrane for testing. The derivatization procedure of the sample is the same as the standard sample solution. 

### 3.6. Instrumentation and Analytical Conditions

The chromatographic system was an Agilent 1260 High-Performance Liquid Chromatography system equipped with an ultraviolet visible detector (HPLC-UV, Agilent Technologies, Santa Clara, CA, USA) operating at 254 nm. LC separations were performed using a ZORBAX Eclipse XDB C_18_ column (4.6 mm×250 mm, 5 μm) maintained at 30 ℃ operating with gradiente elution (0→3 min, 60% A, 40% B; 3→22 min, 85% A, 15% B; 22→25 min, 100% A, 0% B; 25→32 min, 60% A, 40% B; 32→37 min, 60% A, 40% B; 37→40 min, 20% A, 80% B), where (A) is 0.01 mol/L ammonium acetate solution containing 0.1% acetic acid/acetonitrile (*1:9 v/v*) and (B) is 0.01 mol/L ammonium acetate solution containing 0.1% acetic acid/acetonitrile (*v:v 9:1*). The flow rate was 0.8 mL/min and injection volume was 10 µL.

### 3.7. Method Validation

In this study, the standard curve was obtained with the mass concentration of BAs in standard series solutions (1.0~50.0 μg/mL) as the abscissa, and the corresponding peak area as the ordinate. This method is used for analysis verification and quality control by adding 10.0 mg/kg, 50.0 mg/kg and 100.0 mg/kg, respectively, to blank samples for the standard addition recovery test. Each spiked concentration was measured 6 times in parallel, and the average spiked recovery (%) and relative standard deviation (RSD%) were calculated. The following parameters were evaluated by binary linear regression: calibration curve, LOD, LOQ, accuracy (recovery%) and precision (RSD%).

### 3.8. Statistical Analysis

Database entry and statistical analysis used SPSS V21.0 and Microsoft Excel 2007, which are usable to measure continuous summative descriptive indicators and demonstrated by computer. Based on the theory of chemical statistics in analytical chemistry, the classification statistical model of target components is carried out on the basis of sampling statistical analysis. The optimal determination method is optimized and determined, and the objective of batch sample detection and statistical analysis is finally completed. Data were assessed by descriptive statistics. A linear relationship was established using a linear regression model to correlate the BAs concentration and the intensity of the signal analyte.

## 4. Conclusions

Based on the new carbon material–carbon spheres QuEChERS rapid pretreatment combined with HPLC quantitative detection technology, this study detected pollution data of seven BAs obtained from 131 canned sea fish samples on the market and established a food safety risk assessment model. Combined with the consumption data of Chinese residents’ marine fish products, a comprehensive risk assessment of BAs in different regions, different age stages and different genders across the country was conducted. The scientific conclusions of this study have beneficial reference value for government departments to establish aquatic product risk assessment technology, formulate scientific and efficient risk management measures, and reduce the dietary intake risk of biogenic amines in marine fish products of diverse populations. Because fish samples are rich in protein and fat, it is easy to cause a matrix effect to affect the experiment. Liquid quality is not only expensive, but it is also difficult to avoid the interference caused by the matrix effect. However, the matrix effect is hardly considered in this method, and it is completely within the linear range and detection limit, which can meet the actual sample determination.

However, due to the limitation of the evaluation method, this study is only a simple point evaluation model based on specific assumptions and a small number of sample databases. In addition, human toxicology experimental data of BAs are not perfect, the selected total BAs vulnerability is only available in some reports, not domestic and foreign standards, which lead to large errors in the results and the difficulty of risk assessment of BAs. Therefore, the evaluation method has definite uncertainty, and the data of this study can only be used as a reference. Here, it is suggested that international authoritative departments should strengthen the updating of the detection methods of BAs in aquatic products and formulate feasible standards for BAs as soon as possible, so as to scientifically serve the food safety risk assessment.

## Figures and Tables

**Table 1:** Analytical performance of biogenic amines by HPLC-UV.

NO.	BAs	Abb	Time (min)	Curve	R^2^	LOD (μg/kg)	LOQ (μg/kg)	Recovery (%)	RSD (%)
1	Tryptamine	Try	13.114	y = 39.488x + 18.55	0.9997	10.8	36	95.6	1.9
2	Putrescine	Put	19.376	y = 6.659x + 18.78	0.9996	10.8	36	92.3	3.7
3	Cadaverine	Cad	20.991	y = 33.069x + 55.44	0.9998	7.2	24	93.1	4.8
4	Histamine	His	21.882	y = 133.1x + 18.49	0.9999	7.2	24	97.7	2.3
5	Tyramine	Tyr	27.353	y = 96.95x + 11.41	0.9998	10.8	36	94.2	3.2
6	Spermidine	Spd	28.494	y = 89.147x + 29.56	1	7.2	24	92.6	4.1
7	Spermine	Spm	32.142	y = 94.954x + 10.05	1	7.2	24	93.5	2.6

**Table 2:** Average value (mg/kg) and detection range (%) of 7 BAs in eight categories of canned sea fish samples (*n* = 6).

Type	Tryptamine		Putrescine		Cadaverine		Histamine		Tyramine		Spermidine		Spermine		∑7 BAs	
	Mean	Range	Mean	Range	Mean	Range	Mean	Range	Mean	Range	Mean	Range	Mean	Range	Mean	Range
Tunas	ND	ND	29.34	ND~142.63	7.51	ND~118.36	19.59	ND~167.02	6.98	ND~75.97	1.92	ND~19.74	1.46	ND~18.77	66.81	ND~231.05
Sardine	1.22	ND~29.45	27.99	ND~117.81	20.80	ND~165.82	29.69	ND~122.45	28.66	ND~115.60	2.60	ND~26.12	1.31	ND~17.63	112.27	ND~274.49
Anchovy	0.75	ND~18.10	24.09	ND~89.97	21.52	ND~153.63	30.50	ND~75.47	18.41	ND~87.25	1.60	ND~19.17	1.03	ND~16.19	97.91	ND~292.47
Mackerel	ND	ND	21.74	ND~82.80	25.84	ND~123.15	23.07	ND~95.62	12.62	ND~50.64	ND	ND	0.80	ND~11.26	84.07	ND~207.65
Spanish mackerel	ND	ND	9.76	ND~29.10	5.53	ND~33.20	85.44	29.40~282.50	9.25	ND~23.70	10.21	ND~46.80	1.59	ND~9.52	121.77	29.40~368.50
Chub mackerel	ND	ND	31.49	ND~104.03	14.16	ND~70.80	17.75	ND~59.13	25.70	ND~83.00	5.32	ND~26.60	2.71	ND~13.53	97.12	ND~235.84
Saury	ND	ND	24.85	ND~99.40	ND	ND	11.58	ND~46.30	6.85	ND~27.40	ND	ND	ND	ND	43.27	ND~126.80
Bream	ND	ND	9.12	ND~27.37	ND	ND	42.67	12.00~82.36	20.17	ND~41.43	9.14	ND~27.41	4.11	ND~12.32	85.21	61.06~123.79
Average	0.45	ND~29.45	25.81	ND~142.63	15.14	ND~165.82	27.74	ND~282.50	16.25	ND~115.60	2.44	ND~46.80	1.34	ND~18.77	89.18	ND~368.50

Note: ND means not detected.

**Table 3:** Detection rate (Dr, %) and quartile of 7 BAs in eight categories of canned sea fish samples (*n* = 6).

Type	Tryptamine		Putrescine		Cadaverine		Histamine		Tyramine		Spermidine		Spermine		∑7 BAs	
	Dr	P50/P25/P75	Dr	P50/P25/P75	Dr	P50/P25/P75	Dr	P50/P25/P75	Dr	P50/P25/P75	Dr	P50/P25/P75	Dr	P50/P25/P75	Dr	P50/P25/P75
Tunas	ND	ND/ND/ND	54.76	21.20/ND/51.04	14.29	ND/ND/ND	59.52	13.60/ND/26.30	14.29	ND/ND/ND	14.29	ND/ND/ND	11.90	ND/ND/ND	83.33	64.97/17.96/87.23
Sardine	6.06	ND/ND/ND	51.52	11.04/ND/51.91	30.30	ND/ND/31.99	72.73	23.40/ND/41.62	57.58	18.63/ND/54.17	15.15	ND/ND/ND	9.09	ND/ND/ND	84.85	86.84/41.26/195.53
Anchovy	4.17	ND/ND/ND	50.00	6.06/ND/48.71	29.17	ND/ND/22.77	87.50	19.14/13.80/60.86	45.83	12.32/ND/33.93	12.50	ND/ND/ND	8.33	ND/ND/ND	87.50	93.72/15.80/141.29
Mackerel	ND	ND/ND/ND	57.14	17.51/ND/34.39	35.71	ND/ND/57.07	57.14	12.31/ND/43.47	42.86	ND/ND/25.58	ND	ND/ND/ND	7.14	ND/ND/ND	85.71	75.50/26.66/141.40
Spanish mackerel	ND	ND/ND/ND	50.00	6.39/ND/16.66	16.67	ND/ND/ND	100	51.75/29.40/67.80	50.00	6.83/ND/18.11	33.33	ND/ND/14.49	16.67	ND/ND/ND	100	45.10/87.22/113.17
Chub mackerel	ND	ND/ND/ND	40.00	ND/ND/53.40	20.00	ND/ND/ND	40.00	ND/ND/29.60	60.00	ND/ND/25.90	20.00	ND/ND/ND	20.00	ND/ND/ND	80.00	43.13/19.60/187.03
Saury	ND	ND/ND/ND	25.00	ND/ND/ND	ND	ND/ND/ND	25.00	ND/ND/ND	25.00	ND/ND/ND	ND	ND/ND/ND	ND	ND/ND/46.30	50.00	23.15/ND/46.30
Bream	ND	ND/ND/ND	33.33	ND/ND/27.37	ND	ND/ND/ND	100	33.65/11.99/82.36	66.67	ND/ND/27.41	33.33	ND/ND/12.32	33.33	70.77/61.06/123.79	100	58.44/33.65/123.79
Average	2.29	ND/ND/ND	51.15	11.04/ND/46.61	22.90	ND/ND/ND	69.47	16.72/ND/36.41	40.46	ND/ND/24.50	12.98	ND/ND/ND	10.69	ND/ND/ND	83.97	74.68/21.40/121.85

Note: ND means not detected.

**Table 4:** Dietary exposure and food safety index of total biogenic amines.

Type	Age	Gender	Exposure (μg·kg^−1^·d^−1^ )			Food Safety Index (IFS*c*)		
			Nationwide	City	Countryside	Nationwide	City	Countryside
Infant	2~3	Male	209.52	214.99	163.42	0.0195	0.0200	0.0152
Infant	2~3	Female	120.97	144.30	83.75	0.0113	0.0135	0.0078
Children	4~10	Male	115.43	116.31	99.20	0.0108	0.0108	0.0092
Children	4~10	Female	130.43	133.14	90.72	0.0122	0.0124	0.0085
Immaturity	11~17	Male	93.32	98.51	60.76	0.0087	0.0092	0.0057
Immaturity	11~17	Female	70.19	76.44	49.81	0.0065	0.0071	0.0046
Youth	18~44	Male	67.03	69.08	59.33	0.0062	0.0064	0.0055
Youth	18~44	Female	72.23	73.86	67.24	0.0067	0.0069	0.0063
Middle age	45~59	Male	69.72	69.66	66.43	0.0065	0.0065	0.0062
Middle age	45~59	Female	74.09	73.56	73.98	0.0069	0.0069	0.0069
Old age	>60	Male	83.04	73.87	99.86	0.0077	0.0069	0.0093
Old age	> 60	Female	90.45	86.49	96.47	0.0084	0.0081	0.0090

## Data Availability

The data presented in this study are available on request from the corresponding author.
